# Potentiation of synaptic transmission in Rat anterior cingulate cortex by chronic itch

**DOI:** 10.1186/s13041-016-0251-1

**Published:** 2016-07-29

**Authors:** Ting-Ting Zhang, Feng-Yan Shen, Li-Qing Ma, Wen Wen, Bin Wang, Yuan-Zhi Peng, Zhi-Ru Wang, Xuan Zhao

**Affiliations:** 1 Department of Anesthesiology and Intensive Care Medicine, Xinhua Hospital, College of Medicine, Shanghai Jiaotong University, Shanghai, 200092 China; 2 Department of Anesthesiology, Huashan Hosptital, Fudan University, Shanghai, 200040 China; 3 Institute of Brain Functional Genomics, East China Normal University, Shanghai, 200062 China

**Keywords:** Chronic itch, Anterior cingulate cortex, Synaptic transmission, Silent synapse

## Abstract

Itch and pain share similar mechanisms. It has been well documented that the anterior cingulate cortex (ACC) is important for pain-related perception. ACC has also been approved to be a potential pruritus-associated brain region. However, the mechanism of sensitization in pruriceptive neurons in the ACC is not clear. In current study, a chronic itch model was established by diphenylcyclopropenone (DCP) application. We found that both the frequency and amplitude of miniature excitatory postsynaptic currents in the ACC were enhanced after the formation of chronic itch. The paired-pulse ratio in ACC neurons recorded from the DCP group were smaller than those recorded in control group at the 50-ms interval. We also observe a significant increase in the AMPA/NMDA ratio in the DCP group. Moreover, an increased inward rectification of AMPARs in ACC pyramidal neurons was observed in the DCP group. Interestingly, the calculated ratio of silent synapses was significantly reduced in the DCP group compared with controls. Taken together, we conclude that a potentiation of synaptic transmission in the ACC can be induced by chronic itch, and unsilencing silent synapses, which probably involved recruitment of AMPARS, contributed to the potentiation of postsynaptic transmission.

## Introduction

Itch is an uncomfortable sensation and emotional experience that strongly evokes a desire to scratch [1]. Itch is caused by not only skin disease but also systemic, neuropathic, psychogenic and cutaneous disorders [2]. It is well known that itch can be induced through histamine dependent and histamine-independent pathways [3, 4], and both types of itch have been found to activate spinothalamic tract neurons [5, 6]. Neuroimaging studies in humans have confirmed the anterior cingulate cortex (ACC), along with other cortical structures, are activated by itch [7, 8]. Moreover, Descalzi et al. showed that itching enhanced spontaneous excitatory post-synaptic currents in ACC pyramidal neurons [9].

Itch can be classified as either acute or chronic according to the course duration. Acute itch is a daily experience that can usually be abolished by briefly scratching near the area of itching. Chronic itch can be debilitating, and local scratching often provides little relief and can even exacerbate the problem instead [10]. To the best of our knowledge, few studies have focused on itch-related cortical changes in chronic itch.

It is generally known that glutamate-mediated 2-amino-3-(3-hydroxy-5-methyl-isoxazol-4-yl) propanoic acid receptor (AMPAR) functions contribute to excitatory post synaptic transmission. Membrane trafficking of AMPARs is dynamic, and such dynamic trafficking is important for the expression of synaptic plasticity [11, 12]. The electrophysiological characteristics of AMPARs have been investigated using conventional methods that assess the changes in synaptic plasticity.

In the present study, we combined behavioral and electrophysiological approaches to investigate whether there are changes in synaptic plasticity in the ACC during chronic itch. Furthermore, we sought to determine the underlying mechanisms of such changes in synaptic plasticity.

## Methods

### Animals

Young adult (3–5 weeks old) Sprague-Dawley rats were raised in the Animal Center of Key Laboratory of Brain Genomics at East China Normal University. They were housed in a vivarium (temperature: 22–24 °C; humidity: 50–60 %) with a 12-h light/dark cycle and a sufficient food and water supply. All experiments were performed under protocols approved by the Institutional Animal Care and Use Committee at Shanghai Jiaotong University.

### Modeling and behavioral test

**Fig. 1:**
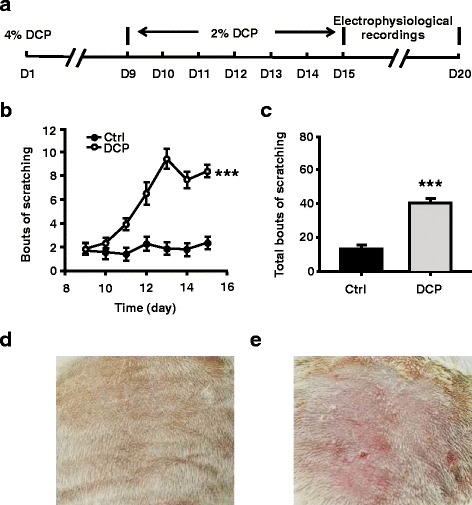
DCP evoked persistent scratching behavior. **a** Schematic of experiment protocol. **b** Scratching behavior in seven consecutive days of challenging. Filled circles indicate bouts of scratching from control rats (*n* = 24 rats); opened circles indicate bouts of scratching from chronic itch rats (*n* = 28 rats). **c** Total bouts of scratching in 7 days. **d** An example of the acetone application site from a control rat. The skin is smooth and the color is a bit dull. **e** An example of the DCP application site from a chronic itch rat. The skin is congestive and scleroid, and accompanied with scratches. ***p < 0.001. D = day; Ctrl = control; DCP = diphenylcyclopropenone

Chronic itch was simulated in accordance with the method described by Sun et al. [13]. Diphenylcyclopropenone (DCP) was dissolved in acetone for reserve. Before DCP application, the skin on the rat’s buttock (~4 cm^2^) was exposed by shaving the hair. DCP (4 %, 0.5 ml) was applied to the exposed skin for sensitization on Day 1 (D1). Seven days after the first application, rats were challenged by applying 2 % DCP (0.5 ml) at the same site once daily until D15. Five minutes after applying DCP, the number of scratching behaviors toward the application site was counted over a 30-min period (Fig. 1a).

On each day of testing, rats were placed in a small transparent plastic chamber (21.5 × 21.5 × 12.5 cm) and were allowed 30 min to acclimate prior to DCP application. One bout of scratching was defined as lifting of a forelimb toward the application site and then returning the forelimb back to the floor, regardless of the number of scratching strokes that were performed between these two movements. Scratching behavior was observed by an investigator blinded to the treatment groups.

### Brain slice preparation

Coronal brain slices of the ACC were prepared following the procedures reported in our previous work [14]. Rats were anesthetized by sodium pentobarbital (80 mg/kg, i.p.) and decapitated on D16-D22, after the behavioral tests were completed. The brain was rapidly excised from the skull and placed in ice-cold artificial cerebrospinal fluid (ACSF) solution (in mM: 119 NaCl, 2.5 KCl, 2.5 CaCl_2_, 1.3 MgSO_4_, 11.0 D-Glucose, 1.0 NaH_2_PO_4_, 26.2 NaHCO_3_) bubbled with carbogen (95 % O_2_ + 5 % CO_2_). One or two minutes later, coronal slices (350 μm) of the ACC were cut from the brain in ice-cold ACSF using a Vibroslicer (VT1000 S, Leica Microsystems, USA). Then, the slices were transferred to the ACSF, which was oxygenated with carbogen at room temperature for incubation. At least 1 h later, the slices were examined in a recording chamber, which was bubbled with carbogen, and the temperature in the chamber was maintained at 28–30 °C. Neurons were visualized using a microscope (DX50WI, Olympus, Japan) via differential interference contrast optics and infrared video microscopy.

### Whole-cell patch-clamp recordings

Standard whole-cell voltage-clamp recordings from the ACC were performed with a MultiClamp 700B amplifier (Axon Instruments, USA) and digitized at 5 kHz by a data acquisition card (DigiData 1440, Axon Instruments, USA) in all electrophysiological experiments. The series resistance (15–30 MΩ) was monitored during the entire recording process. Data were discarded if the series resistance changed by more than 20 % during a single recording. The micropipettes (3.5–6.5 MΩ) were pulled from borosilicate glass (1.5 mm outer diameter and 1.1 mm inner diameter; Sutter Instruments, USA) using a P-97 (Sutter Instruments, USA) and were filled with an internal solution containing (in mM) 125.0 K-gluconate, 10.0 Na_2_-phosphocreatine, 2.0 KCl, 0.5 EGTA, 10.0 HEPES, 4.0 Mg-ATP, 0.3 Na_3_GTP (adjusted to pH of 7.2 with KOH). Miniature excitatory postsynaptic currents (mEPSCs) were recorded from ACC neurons located in cortical layers II/III, and the neurons were voltage clamped at -70 mV in the presence of tetrodotoxin (TTX, 0.5 μM). The extracellular solution routinely contained picrotoxin (0.1 mM) to blockγ-aminobutyric acid A receptor(GABA_A_R) mediated currents except in the recording of miniature inhibitory postsynaptic currents (mIPSCs). For investigating an obvious GABAergic transmission at resting condition (-70 mV), we used the internal solution with a high Cl^-^ concentration, which contained (in mM) 11 EGTA, 2 MgCl_2_, 1 CaCl_2_, 10 HEPES, 115 K-Glu, 2 K_2_ATP, 25 KCl (adjusted to pH of 7.2 with KOH). It has been proved that high intracellular concentrations of Cl^-^ would not be altering resting cell properties [15]. The perfusion solution contained TTX (0.5 μM), 2-amino-5-phosphonopentanoate (AP5, 50 μM) and 6,7-dinitroquinoxaline-2,3(1H,4H)-dione (DNQX, 20 μM).

To elicit synaptic responses from ACC neurons located in cortical layers II/III, stimulation was delivered via a bipolar tungsten electrode placed in layer V of the ACC. The amplitude of the evoked excitatory postsynaptic currents (EPSCs) was adjusted within 30–100 pA at the holding potential of -70 mV in the presence of AP5 (50 μM), except during the experiment in which the AMPA-to-NMDA (N-methyl-D-aspartate) receptor ratio was measured. In the paired-pulse ratio (PPR) experiments, the intervals were 50, 75, 100, 150 ms. For experiments in which AMPA-to-NMDA receptor ratio was measured, the intracellular solution contained (in mM) 115.0 Cs-gluconate, 8.0 NaCl, 20.0 HEPES, 5.0 TEA-Cl, 0.2 EGTA, 0.3 Na_3_GTP, and 4.0 Mg-ATP (adjusted to pH of 7.2 with CsOH). AMPAR-mediated synaptic responses were measured at -70 mV, whereas NMDAR-mediated responses were measured at +40 mV and at a latency where AMPAR responses had fully decayed (60 ms) [16]. Synaptic responses were averaged over 50–100 trials. For calculating the rectification of AMPA receptor-mediated EPSCs, the internal solution consisted of the following (in mM): 140.0 cesium methanesulfonate, 5.0 NaCl, 0.5 EGTA, 10.0 HEPES, 0.1 Na_3_-GTP, 2.0 Mg-ATP, 0.1 spermine, 2.0 QX314, and 10.0 phosphocreatine disodium (adjusted to pH of 7.2 with CsOH). We recorded current at the holding potentials of +35 and -65 mV and used the ratio of the peak amplitude of EPSCs at negative (-65 mV) and positive (+35 mV) holding potentials as the rectification index [17].

Failure rate experiments were performed using the minimum stimulation, as described previously [18, 19]. Cs^+^-based internal solution, which contained (in mM) 115.0 Cs-gluconate, 8.0 NaCl, 20.0 HEPES, 5.0 TEA-Cl, 0.2 EGTA, 0.3 Na_3_GTP, 4.0 Mg-ATP, and 2.0 QX314 (adjusted to pH 7.2 with CsOH), was used. Stimulation with 2-sec intervals were produced using a pulse generator (Master-8, A.M.P. Instruments, Israel) and through a stimulus isolator (ISO-Flex, A.M.P. Instruments, Israel). The stimulus intensity was first identified around the threshold stimulus. The stimulus intensity was then adjusted until the percentage at which the stimulation failed to evoke a response, or the failure rate, was approximately 50 % at the holding voltage of -65 mV. We used this final stimulus intensity to evoke responses in the same cell at the holding voltage of +40 mV. For each cell, 200–300 sweeps were recorded at -65 mV and +40 mV. The failure rate was defined as the percentage of failed evoked responses over sweeps with holding potentials of -65 and +40 mV. All failure responses were visually checked and verified. The fraction of silent synapses was calculated using the equation 1-ln(F_-65_)/ln(F_+40_) as described previously [19]. F_-65_ was defined as the failure rate at -65 mV, whereas F_+40_ was defined as the failure rate at +40 mV.

### Data analysis

The results are shown as the mean ± SEM. Statistical significance was assessed using Student’s *t* test when two groups were compared or a paired t-test when scores before and after an intervention were compared within the same group. The Mann-Whitney rank sum test was used for nonparametric tests. A two-way ANOVA was performed with group as a between-group factor and time as a repeated-measures factor. ANOVA was followed by the Bonferroni *post hoc* test. In all experiments, *p* < 0.05 was considered statistically significant.

## Results

### DCP evoked persistent scratching behavior

Sun et al. reported that DCP could induce persistent scratching behavior in mice [13]. Following their protocol, we found that DCP could induce persistent scratching behavior in rats as well (two-way ANOVA followed by Bonferroni *post hoc* test, *p* < 0.001; Fig. 1b). The DCP group showed significantly more scratching compared with the control group during the 7 days of treatment (Control group: 13.21 ± 1.26 bouts, *n* = 24 rats; DCP group: 40.14 ± 2.63 bouts, *n* = 28 rats, Mann-Whitney, rank sum test, *p* < 0.001; Fig. 1c). These results indicated that a chronic itch model was successfully established by daily application of DCP.

### Potentiation of mEPSCs in ACC neurons in the chronic itch model

**Fig. 2:**
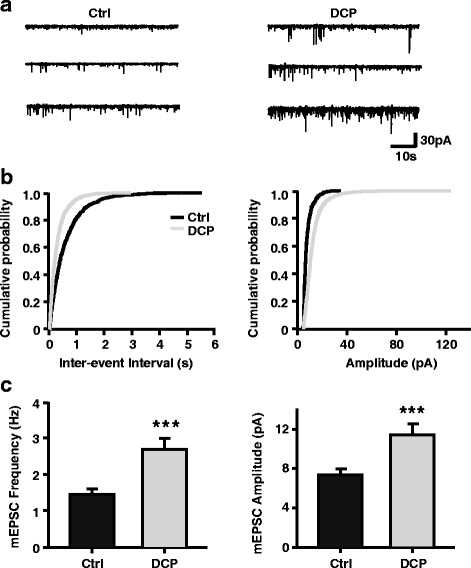
Potentiation of mEPSCs in ACC neurons of chronic itch animal model. **a** Representative mEPSCs recorded from pyramidal neurons in the layer II/III of ACC in control rats (left) and in the rats with chronic itch (right) at a holding potential of -70 mV. Scale bars represent 30 pA and 10 s. **b** Cumulative interval (left) and amplitude (right) histograms of mEPSCs from control rats (*black*, *n* = 12 neurons) and chronic itch rats (*grey*, *n* = 12 neurons). **c** Summary data of mEPSC frequency (left) and amplitude (right). ***p < 0.001. mEPSC = miniature excitatory postsynaptic current; ACC = anterior cingulate cortex; Ctrl = control; DCP = diphenylcyclopropenone

Human imaging studies showed that the ACC is active simultaneously with itch [7, 8]. Therefore, to explore the potential mechanisms of itching behavior, we assessed ACC neuronal activity in vitro using whole-cell patch clamp recordings. Each rat in which chronic itch was successfully induced was sacrificed 5 days after all behavioral tests were completed. We recorded mEPSCs of pyramidal neurons in layers II/III of the ACC, and found an obvious increase in the frequency of mEPSCs in the DCP group compared with the control group (Control: 1.56 ± 0.14 Hz, *n* = 12 neurons/5 rats; DCP: 2.87 ± 0.30 Hz, *n* = 12 neurons/5 rats, *p* < 0.001, *t* test; Fig. 2b, c). Moreover, the mean amplitude of mEPSCs in the DCP group was much higher than that in the control group (Control: 7.89 ± 0.34 pA, *n* = 12 neurons/5 rats; DCP: 12.22 ± 0.93 pA, *n* = 12 neurons/5 rats, *p* < 0.001, Mann-Whitney Rank Sum Test; Fig. 2b, c).

**Fig. 3:**
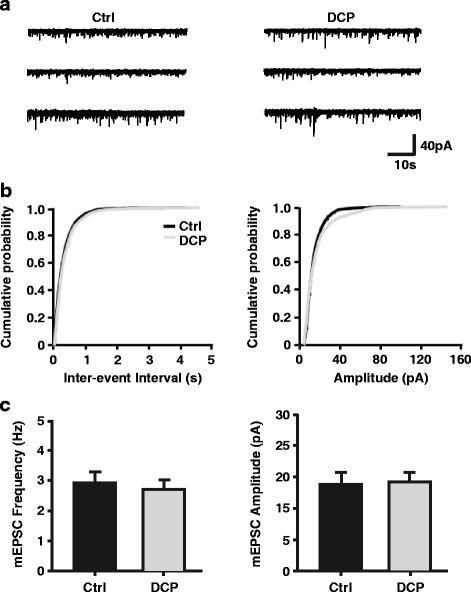
No change in mIPSCs in ACC neurons of chronic itch animal model. **a** Representative mIPSCs recorded from pyramidal neurons in the layer II/III of ACC in control rats (left) and in the rats with chronic itch (right) at a holding potential of -70 mV. Scale bars represent 40 pA and 10 s. **b** Cumulative interval (left) and amplitude (right) histograms of mIPSCs from control rats (*black*, *n* = 9 neurons) and chronic itch rats (*grey*, *n* = 9 neurons). **c** Summary data of mIPSC frequency (left) and amplitude (right). mIPSC = miniature inhibitory postsynaptic current; ACC = anterior cingulate cortex; Ctrl = control; DCP = diphenylcyclopropenone

We also assessed inhibitory postsynaptic transmission in the ACC after establishing the model. Unlike the mEPSCs, the mean amplitude and frequency of mIPSCs were not significantly different between groups (Control: 3.18 ± 0.38 Hz, *n* = 8 neurons/3 rats; DCP: 2.93 ± 0.29 Hz, *n* = 9 neurons/3 rats, *p* = 0.955, t test; Control: 15.00 ± 1.97 pA; DCP: 15.31 ± 1.34 pA, *p* = 0.983, *t* test; Fig. 3b, c). The intrinsic membrane properties was not changed after the development of chronic itch, since neither the resting membrane potential nor input resistances were different between control and chronic itch group (Control: -71.95 ± 1.66 mV, 104.21 ± 6.58 MΩ, *n* = 12 neurons/3 rats; DCP: -70.96 ± 1.79 mV, 112.54 ± 6.36 MΩ, *n* = 12 neurons/3 rats, *p* > 0.05, *t* test). Based on our results, the increased excitability of ACC pyramidal neurons in chronic itch may be related to enhanced excitatory synaptic transmission in this area.

### Decreased PPR in ACC neurons in the chronic itch model

**Fig. 4:**
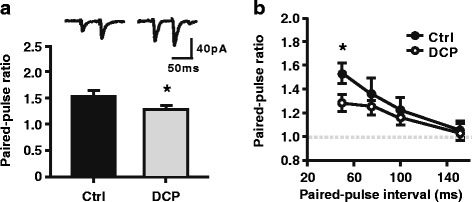
Decreased PPR in ACC neurons of chronic itch model. **a** Up, sample traces of paired EPSCs in response to two sequential presynaptic stimuli at an interval of 50 ms recorded in lay II/III of the ACC. Scale bars represent 40pA and 50 ms. Down, summary of PPR at 50-ms interval. **b** Summary data of PPR in control (filled circles, *n* = 13 neurons) and DCP (opened circles, *n* = 10 neurons) group. *p < 0.05. PPR = paired-pulse ratio; ACC = anterior cingulate cortex; EPSC = excitatory postsynaptic current; Ctrl = control; DCP = diphenylcyclopropenone

The increase in mEPSCs frequency is considered to reflect facilitation of presynaptic transmission [17]. In the current study, we used the method of PPR to clarify the change in presynaptic transmission in the ACC of a chronic itch model. PPR is a transient form of plasticity that is commonly used as a measure of presynaptic function. In PPR, two pulses are delivered, and the response to the second stimulus is enhanced as a result of residual calcium in the presynaptic terminal after the first stimulus [20]. At the 50-ms interval between pulses, we found that the PPR in ACC neurons recorded from rats in the chronic itch group were obviously smaller than those recorded from the control group (Control: 1.53 ± 0.08, *n* = 13 neurons/5 rats; DCP: 1.27 ± 0.04, *n* = 10 neurons/4 rats, *p* < 0.05, Mann-Whitney rank sum test; Fig. 4). These results suggest that a presynaptic mechanism is involved in the enhanced excitatory synaptic transmission in layers II/III of the ACC after the formation of chronic itch.

### Upregulation of AMPAR in ACC neurons in the chronic itch model

**Fig. 5:**
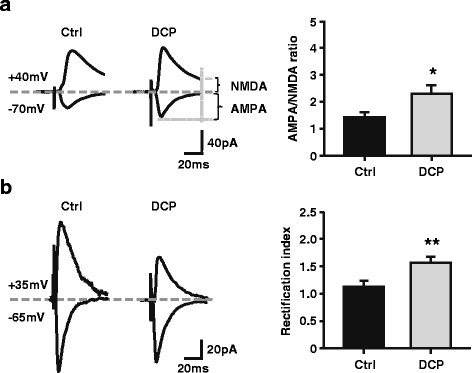
Altered AMPA/NMDA ratio and rectification index of AMPAR-mediated currents in the ACC after the formation of chronic itch. **a** Left, sample traces of evoked synaptic AMPA and NMDA currents recorded at -70 and +40 mV respectively. Scale bars represent 40 pA and 20 ms. Right, summary data of AMPA/NMDA ratio in control (*black*, 12 neurons) and DCP (*grey*, 12 neurons) group. AMPAR-mediated synaptic responses were measured at -70 mV, whereas NMDAR-mediated responses were measured at +40 mV and at a latency where AMPAR responses had fully decayed (60 ms). *p < 0.05. **b** Left, sample traces of evoked AMPAR-mediated postsynaptic currents at -65 and +35 mV holding potentials. Right, Summary data of rectification of AMPAR-mediate currents in control (*black*, 11 neurons) and DCP (*grey*, 18 neurons) group. Scale bars represent 20 pA and 20 ms. **p < 0.01. ACC = anterior cingulate cortex; Ctrl = control; DCP = diphenylcyclopropenone

The AMPAR is a major excitatory glutamate receptor in the postsynaptic membrane [21]. To address whether a postsynaptic mechanism was involved in the enhancement of excitatory glutamatergic transmission, we examined the AMPA/NMDA ratio and rectifying properties of AMPARs in ACC pyramidal neurons. As shown in Fig. 5a, we observed a significant increase in the AMPA/NMDA ratio in the DCP group (Control: 1.43 ± 0.18, *n* = 12 neurons/4 rats; DCP: 2.30 ± 0.31, *n* = 12 neurons/5 rats, *p* < 0.05, Mann-Whitney rank sum test; Fig. 5a). Furthermore, we found an increased inward rectification of AMPARs in ACC pyramidal neurons in the chronic itch group (Control: 1.12 ± 0.08, *n* = 11 neurons/6 rats; DCP: 1.57 ± 0.09, *n* = 18 neurons/7 rats, *p* < 0.01, *t* test; Fig. 5b). These results indicate that a postsynaptic mechanism also contributed to the enhancement of excitatory glutamatergic transmission.

### Unsilencing of silent synapses in ACC neurons in the chronic itch model

**Fig. 6:**
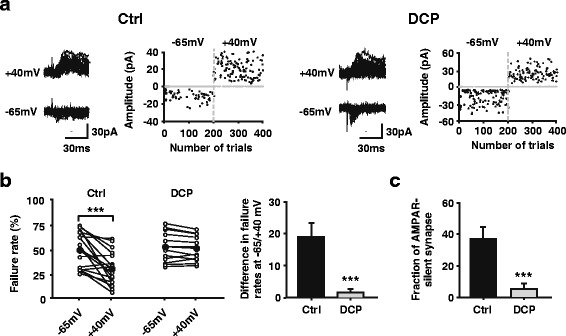
Reduction in silent synapses after the formation of chronic itch. **a** Representative consecutive traces and scatter plot of evoked EPSCs at -65 and +40 mV holding potentials from one pyramidal neuron in ACC from control (left) and DCP group (right). The amplitude of failed events are assigned to 0 pA for ease of visualization. Scale bars represent 30 pA and 30 ms. **b** Left, quantification of failure rate of evoked responses for control (*n* = 12 neurons) and DCP (*n* = 16 neurons) group at -65 and + 40 mV holding potentials. The failure rate difference between -65 and +40 mV decreased after the formation of chronic itch. ***p < 0.001. Right, failure rate difference of evoked responses between -65 and +40 mV holding potentials is obviously lower from chronic itch rats than controls. **p < 0.01. **c** The percentage of silent synapses is reduced after chronic itch compared with controls. **p < 0.01. EPSC = excitatory postsynaptic current; ACC = anterior cingulate cortex; Ctrl = control; DCP = diphenylcyclopropenone

The AMPA/NMDA ratio is thought to provide a rough indication of AMPA-silent synapses in the population [22]. It was shown previously that some glutamatergic synapses lack AMPARs and contain only NMDARs, and these synapses are functionally silent even though presynaptic glutamate release is normal [23]. Silent synapses were found almost everywhere in the brain [24]. Silent synapses are defined by a small failure rate of evoked EPSCs (eEPSCs) at positive potentials (+40 mV) concurrent with a large failure rate to the same stimuli at negative potentials (-65 mV) [19, 25]. We found that the failure rate, measured at +40 mV, was significantly decreased compared with that at -65 mV in pyramidal neurons recorded from layers II/III of the ACC in control rats (-65 mV: 49.25 ± 4.53 %; +40 mV: 30.40 ± 4.18 %, *n* = 16 neurons/8 rats, *p* < 0.001, paired *t* test; Fig. 6a, b). This result indicates the presence of silent synapses in the ACC of young adult rats. In contrast, the failure rate was comparable between the negative and positive holding potentials in the DCP group (-65 mV: 50.85 ± 4.24 %; +40 mV: 49.21 ± 3.53 %, *n* = 12 neurons/9 rats, *p* = 0.154, paired t test; Fig. 6a, b), indicating that most synapses are activated in the ACC after chronic itch. Accordingly, the calculated ratio of silent synapses was significantly reduced in the DCP group (5.38 ± 3.18 %, *n* = 12 neurons/9 rats) compared with controls (36.96 ± 7.36 %, *n* = 16 neurons/9 rats, *p* < 0.01, Mann-Whitney rank sum test; Fig. 6c). These results indicate that the silent synapses were unsilenced by inserting AMPARs into the postsynaptic membrane after chronic itch.

## Discussion

Chronic itch can be induced by peripheral neuropathy or nerve irritation [26]. According to previous studies, itch and pain share similar mechanisms [3]. These conditions can result from immune dysfunction because inflammatory mediators can directly activate or sensitize nociceptive and pruriceptive neurons in the peripheral and central nervous systems, leading to pain and itch hypersensitivity [27]. DCP acts as a local irritant, triggering a local sensitization, and it triggers an immune response that opposes the action of autoreactive cells [28]. In our study, we found that DCP application can induce chronic itch in a rat model.

The ACC is critical for cognitive functions, including decision making, trace memory, attention, and persistent pain [29, 30]. It has been well documented that the ACC is important for pain-related perception, and there are both presynaptic and postsynaptic changes in ACC synapses after nerve injury [17, 31]. Long-term changes in synaptic transmission, occurring in sensory synapses located along the somatosensory pathway or pain-processing brain regions, contribute to chronic inflammatory and neuropathic pain [32–35]. Therefore, we explored the synaptic transmission of the ACC in a chronic itch model. Our results showed that the enhanced excitatory synaptic transmission in the ACC after chronic itch can be attributted to increases in the probability of presynaptic neurotransmitter release and postsynaptic responsiveness.

AMPA receptors are heteromultimers assembled from GluR1, GluR2, GluR3 and GluR4 subunits [36, 37]. AMPA receptors without GluR2 are Ca^2+^ permeable (CP-AMPA receptors) and inwardly rectifying, which are always GluR1 heteromultimer [38]. Therefore, an alteration in the rectification index could reflect the subunit composition of AMPA receptors. In previous studies, the number of synaptic GluR1 subunits in the ACC of rats increased with nerve injury and chronic inflammation pain [17, 39]. The trafficking of AMPA receptor subunits has been proposed to contribute to synaptic plasticity underlying hyperalgesia [32]. In our study, we found an intensified function of CP-AMPARs in ACC neurons after the development of chronic itch. This result indicates that, similar to neuropathic pain, the dynamic membrane trafficking of CP-AMPAR in ACC is also induced by chronic itch.

Long-term synaptic plasticity often results in a change in the number of functional synaptic modules connecting pre- and postsynaptic neurons, conversion of silent synapses to functional synapses (unsilencing), and conversion of functional synapses to silent synapses (silencing), which should occur frequently in the brain [22]. Our data suggest that silent synapses were unsilenced by recruiting AMPARs in ACC neurons after chronic itch. Although evidence indicates that AMPA-silent synapses are largely confined to the prepubescent developmental period [22], some AMPA-silent synapses may exist among mature synapses and ageing animals [40]. In the current study, the presence of silent synapses in the ACC of young adult rats (3-5 weeks old) is supported by the reduced failure rate of eEPSCs at depolarized holding potentials. Furthermore, in DCP group, the failure rate of eEPSCs did not differ between positive and negative holding potentials, indicating that silent synapses were unsilenced postsynaptically.

In addition to postsynaptically silent synapses, presynaptically silent synapses also exist. Presynaptically silent synapses do not release neurotransmitter in response to presynaptic action potentials, and they are silent when receiving low-frequency stimulation but begin releasing neurotransmitters, and are therefore not silent, when they receive high-frequency stimulation [41]. We found that there was an increase in the frequency of mEPSCs in ACC pyramidal neurons after chronic itch was induced. Moreover, the PPR of ACC neurons in the chronic itch group was smaller than those in the control group at 50-ms intervals. These results indicate that presynaptically silent synapses were unsilenced as well.

There are some limitations in this study that should be noted. First, NMDAR is another type of major glutamate receptor, and it has been considered a potential target for the treatment of itch [42]. Moreover, NMDAR, especially NR2B, in the ACC plays an important role in the induction and expression of persistent inflammatory and neuropathic pain [43, 44]. The contribution of NMDARs was not estimated in the current study. Second, the rectifying properties of AMPARs can only reveal the function of CP-AMPARs. Whether other subtypes of AMPARs contributed to the chronic itch need to be investigated in the future work. Third, Descalzi et al. showed that the acute scratching corresponds with enhanced excitatory transmission in the ACC through kainate receptor modulation of inhibitory circuitry [9]. However, we found that the GABAergic transmission was not affected by chronic itch. It has been well known that kainate receptors are rare in pyramidal neurons. We therefore never considered the contribution of kainate currents in the electrophysiology experiments. However, kainate receptor GluK1 is considered to be incorporated into the silent synapses in CA1 pyramidal neurons [45]. Thus, whether the redistribution of kainate receptors on ACC pyramidal neurons under the chronic itch condition is curious.

## Abbreviations

ACC, anterior cingulate cortex; ACSF, artificial cerebrospinal fluid; AMPA, 2-amino-3-(3-hydroxy-5-methyl-isoxazol-4-yl) propanoic acid; AP5, 2-amino-5-phosphonopentanoate; DCP, diphenylcyclopropenone; DNQX, 6,7-dinitroquinoxaline-2,3(1H,4H)-dione; EPSCs excitatory postsynaptic currents; GABA, γ-aminobutyric acid; mEPSCs, miniature excitatory postsynaptic currents; mIPSCs, miniature inhibitory postsynaptic currents; NMDA, N-methyl-D-aspartate; PPR, paired-pulse ratio; TTX, tetrodotoxin
